# Clinical knowledge, experiences, and perceptions of yoga providers in arthritis treatment: a UK-based qualitative study

**DOI:** 10.1007/s00296-025-05843-1

**Published:** 2025-04-04

**Authors:** Isha Biswas, Busola Adebusoye, Sarah Lewis, Kaushik Chattopadhyay

**Affiliations:** 1 Lifespan and Population Health, School of Medicine, University of Nottingham, Nottingham, NG5 1PB UK; 2 Centre for Medical Imaging, University College London, London, W1W 7TS UK; 3 The Nottingham Centre for Evidence-Based Healthcare: A JBI Centre of Excellence, Nottingham, NG5 1PB UK

**Keywords:** Arthritis, Interview, Mind–body therapies, Qualitative research, United Kingdom, Yoga

## Abstract

This study explored yoga providers’ clinical knowledge of arthritis, experiences of delivering yoga to people with arthritis, their perceived role in arthritis treatment, and perceived yoga training needs. Qualitative semi-structured interviews with 20 United Kingdom (UK)—based yoga providers were conducted. The interviews were digitally recorded, transcribed verbatim, and analysed thematically. The analysis generated eight themes. Yoga providers were generally aware of their attendees’ health conditions and had clinical knowledge of arthritis through their yoga training. They were reasonably confident in delivering yoga to attendees with arthritis and felt that they had an important role in supporting these attendees. Gentle yoga practices were considered appropriate by the yoga providers, with a perception that a potential interplay between major components of yoga including yogic poses (asana), breathing practices (pranayama), and meditation (dhyana) and relaxation practices could help impart mind–body benefits in arthritis. Creating a safe and supportive environment in yoga sessions, being empathetic towards attendees’ needs, and offering tailored modifications were perceived to be important for delivering yoga in arthritis treatment. Major system-level challenges to yoga delivery in arthritis treatment included the inadequate promotion of yoga, the unregulated nature of yoga delivery, and the absence of evidence-based arthritis-specific yoga training. Yoga providers felt they could play a key role in arthritis treatment in the UK, provided yoga is adequately promoted and made accessible to people with arthritis, yoga delivery is regulated, and arthritis-specific yoga training using the best available scientific evidence is made accessible to them.

## Introduction

Arthritis is a set of acute or chronic musculoskeletal conditions, characterised by warm, swollen, painful, stiff, and deformed joints, and can result in poor joint function [1]. Osteoarthritis and rheumatoid arthritis are the most common forms of arthritis and their global prevalences are expected to increase [2, 3]. Over 10 million people in the United Kingdom (UK) have some form of arthritis, most commonly osteoarthritis, with negative impacts on the affected individual, family, society, and economy [4]. Western medicine primarily treats arthritis with medications like non-steroidal anti-inflammatory drugs (NSAIDs) and disease-modifying anti-rheumatic drugs (DMARDs), plus exercise [5–7]. However, side effects of long-term medications and challenges with adherence to conventional Western exercise lead people to explore alternatives [8, 9].

Originating in the Indian subcontinent, yoga is a comprehensive mind–body discipline offering holistic well-being [10]. Due to yoga’s growing appeal, many people practice it in various health conditions and enhance their well-being, globally [11–17] and in the UK [14–17]. For instance, a UK-based cross-sectional study reported arthritis as one of the most commonly disclosed health conditions in yoga sessions [15]. Several systematic reviews suggest that yoga may be a safe and beneficial practice in addition to standard medical treatment for osteoarthritis and rheumatoid arthritis [18–21]. Additionally, the American College of Rheumatology (ACR) and the European League Against Rheumatism (EULAR) guidelines recommend yoga as a beneficial part of treatment for these conditions [22–24].

In the UK, yoga is delivered by a range of providers with different levels of training [25]. These include around 10,000 yoga teachers [26] and nearly 150 yoga therapists with additional yoga training and experience in delivering yoga for specific health conditions [27]. Most yoga sessions are provided by yoga teachers who may have some clinical knowledge and experience in delivering yoga for health conditions but may benefit from further yoga training to support people with specific health conditions [14].

Despite yoga’s potential benefits in arthritis treatment and its increasing popularity, there is no evidence exploring the clinical knowledge, experiences, or perceptions of yoga providers in arthritis treatment. Yoga providers’ insights as healthcare professionals would help identify and address the potential issues in recommending yoga in arthritis treatment and provide solutions to these challenges. Arthritis encompasses a variety of types, many of which share overlapping symptoms and health consequences, making it challenging to distinguish between them without a confirmed clinical diagnosis [28]. Therefore, to reduce complexity and ensure that yoga providers can deliver effective and inclusive interventions without needing to differentiate between the types of arthritis, this study focused on their insights regarding the delivery of yoga to individuals with any form of the condition. Hence, this study explored UK-based yoga providers’ clinical knowledge of arthritis, experiences of delivering yoga to people with arthritis, their perceived role in arthritis treatment, and perceived yoga training needs.

## Methods

### Study design, philosophical paradigm, research approach, location, year, and reporting

A qualitative study underpinned by the constructivist philosophical paradigm was conducted utilising the phenomenological approach in the UK in 2023 [29, 30]. The study was reported according to the Consolidated Criteria for Reporting Qualitative Research (COREQ‐32) guideline [31].

### Eligibility criteria and participant recruitment

Eligible participants were UK-based yoga providers (teachers and/or therapists) who delivered yoga sessions to people with arthritis aged 18 years or older, at least once a fortnight within the last six months from the recruitment date, in any setting within the UK. Yoga providers were identified using a purposive sampling approach. They were contacted through professional yoga associations in the UK. Briefly, five yoga associations were approached to circulate the study poster with contact details of the lead researcher and PhD student; trained in qualitative research methods (IB), and two agreed namely, British Wheel of Yoga (BWY) and Yoga Teachers Together (YTT) (previously Independent Yoga Network (IYN)). 22 yoga providers were initially interested and contacted the lead researcher but two of them did not respond to the follow-up emails. The participant information sheet and consent form were shared with potential participants via email, and the lead researcher answered their questions if they had any. Before conducting interviews, written informed consent was obtained from each participant.

### Sample size

The sample size guideline for qualitative research using semi-structured interviews suggests that 20 to 30 interviews are adequate for achieving saturation of themes [32]. Although data saturation was achieved on the 17^th^ interview; we continued interviewing until the 20^th^ to ensure no new relevant insights were missing.

### Interview guide

The lead researcher and two senior researchers (SL and KC) developed an interview guide based on the study’s aim and the existing relevant literature. Probing questions were included to encourage the yoga providers to share their opinions freely. For pre-testing, the interview guide was discussed with a UK-trained yoga teacher for her feedback. However, no major adjustments were required.

### Interview procedures and transcription

Once the interview date, time, and mode (online through Microsoft Teams or in-person) were finalised, the interviews were conducted by the lead researcher in English using the interview guide. All interviews were digitally recorded using Microsoft Teams with the participants’ (yoga providers’) permission. In addition, research notes were maintained to facilitate preliminary coding. Participants turned their cameras on during the interviews, which allowed the lead researcher to engage more with the participants and to check their non-verbal expressions for data analysis and interpretation. Participants received a £10 online shopping voucher for their time. Two interview recordings were transcribed verbatim by the lead researcher for familiarisation with the data. An external company transcribed the remaining interview recordings after signing the non-disclosure agreement. The lead researcher repeatedly listened to the interview recordings to add any missing data and correct any inaccuracies. All transcripts were anonymised by removing identifiable information and labelling each transcript with a number.

### Data analysis

Interview transcripts were imported into NVivo 12 software to facilitate data storage and organisation for analysis [33]. Data were analysed using the thematic analysis approach of Braun and Clarke [34]. This followed several steps: (i) familiarisation through multiple readings of the interview transcripts (first five transcripts by two independent researchers (IB and BA/SL) and remaining transcripts by the lead researcher), (ii) line-by-line inductive coding i.e., assigning codes to units within the data (IB and BA/SL), (iii) combining the codes into sub-themes after discussion with senior researchers (IB and SL/KC), and (iv) interpreting and organising the codes into over-arching themes (IB and SL/KC). The themes were refined on two levels—primarily with the coded data ensuring they formed a coherent pattern. Next, the emergent themes were organised in an analytical order such that there was a connection between themes, accurately reflecting the complete data. Each theme was described with anonymised participant quotes that best supported them (represented by “T” and “Th” for yoga teachers and yoga therapists, respectively). Quotes reported were verbatim unless indicated by an ellipsis (…) to indicate that small sections of text were removed for clarity.

### Ethical approval

Ethical approval was obtained from the University of Nottingham’s Faculty of Medicine and Health Sciences Research Ethics Committee (FMHS 40–0722).

## Results

Twenty UK-based yoga providers were interviewed online, including 16 yoga teachers and 4 yoga therapists. Most of them were females (n = 19) aged 45 to 64 years (n = 16). Most of them had a diploma in yoga (n = 16) and their yoga delivery experience ranged from 11 months to over 50 years. The interviews had a mean duration of 43 min, ranging from 27 to 70 min. Yoga providers’ self-reported characteristics are detailed in Table 1.

**Table 1:** Yoga providers’ self-reported characteristics

Characteristics	N
Yoga providers	20
Yoga teacher	16
Yoga therapist	4
Sex	
Female	19
Male	1
Age group, in years	
25–44	2
45–64	16
65 or more	2
Yoga training received^a^	
Certificate	10
Diploma	16
PG certificate	3
PG diploma	3
Other	2
Yoga delivery experience (range)	11 months to over 50 years
Style of yoga^a^	
Hatha yoga	9
Vinyasa yoga	3
Ashtanga yoga	1
Other	10
Major content of yoga sessions^a^	
Asana (yogic poses)	20
Pranayama (breathing practices)	19
Dhyana (meditation) and relaxation practices	20
Structure of yoga sessions^a^	
Group	20
One-on-one	14
Yoga settings^a^	
Yoga class	19
Yoga workshop	6
Attendees’ home	5
Outdoors	6
Other	15
Terminology from other language used in yoga sessions^a^	
Sanskrit (for explaining yoga philosophy and names of yogic practices)	9
Scottish	1

^a^Participants chose more than one option

### Thematic analysis

Eight themes and 22 subthemes were identified (see Table 2). The verbatim quotes are reported in Table 3.

**Table 2:** Themes and subthemes identified

Theme	Subtheme
1. Awareness and clinical knowledge of attendees’ health conditions, including arthritis, and its treatment	1.1 Awareness of health conditions 1.2 Clinical knowledge of arthritis, its treatment, and limitations
2. Experiences in delivering yoga to attendees with arthritis and their perceptions of arthritis treatment shaped by these experiences	2.1 Experiences of modifying yoga for attendees with arthritis 2.2 Perceived confidence level in delivering yoga to attendees with arthritis 2.3 Perceived importance of their role in arthritis treatment
3. Perceived benefits of yoga for attendees with arthritis	3.1 A better alternative for movement compared to other forms of physical activity 3.2 Physical and mental well-being 3.3 Social well being 3.4 Potential role of yoga as a part of arthritis treatment
4. Perceived beneficial components of yoga in arthritis treatment	4.1 Yogic poses help mobilise the joints 4.2 Breathing practices, and meditation and relaxation practices work together to release mental stress 4.3 Yogic poses, breathing practices, and meditation and relaxation practices are interrelated in imparting mind–body benefits in arthritis
5. Perceived importance of the yoga environment and yoga provider qualities in arthritis treatment	5.1 Creating and maintaining a positive environment 5.2 Yoga provider qualities
6. Attendee-level concerns perceived as challenges for yoga delivery in arthritis treatment	6.1 Logistical challenges 6.2 Apprehensions about trying yoga among attendees with arthritis 6.3 Lack of acceptance of physical limitations among attendees with arthritis 6.4 Limited awareness of yoga among attendees with arthritis
7. System-level concerns perceived as challenges for yoga delivery in arthritis treatment	7.1 Unregulated nature of yoga delivery 7.2 Limited available scientific evidence on the use of yoga in arthritis treatment
8. Professional yoga training obtained and perceived yoga training needs in arthritis treatment	8.1 Professional yoga training obtained 8.2 Perceived yoga training needs in arthritis treatment

**Table 3:** Verbatim quotes representing each theme

1a	“You get them to fill in a health questionnaire before they come and see you. on that form…it’s got tick boxes…and then I’ll have a bit of discussion with them…” (T5)
1b	“It’s a range of issues, arthritis, including rheumatoid and osteoarthritis…just general, from cancer to they’ve had strokes, fibromyalgia, chronic fatigue, eye issues, there’s a whole host…” (Th3)
1c	“ …I think people get to an age and think, “I’ve got dodgy knees now” but I don’t think they’ve actually got it formally diagnosed…” (T15)
1d	“…So osteoarthritis is probably the one…the shape of the bone end changes…the cartilage process degeneration and regeneration is sort of disrupted…rheumatoid arthritis is an autoimmune condition so there’s a mechanism where the body itself is attacking the joints…it creates inflammation and swelling and change of shape of joint again, it tends to go through episodes or flare-ups…” (T2)
1e	“The NICE pathway is to first diagnose and then it used to be steroids, prednisolone was the first point of call but I’m seeing people are given hydroxychloroquine and for extreme cases, they give TNF, methotrexate and other biologics which obviously have side effects. When the disease gets into remission, the TNFs are removed and they are on low-dose steroids. They give a lot of steroid shots, injections into the knee before they go into the knee replacement option.” (Th1)
1f	“…I think it’s more symptom control rather than prevention that they tend to promote. So when they’re seeing somebody, they’ve already got a lot of pain and maybe lost function and mobility by the time they’re actually seen by the NHS…” (T13)
2a	“I’ve got chairs for some people if they want to use a chair for balance…if they want to sit on something like blankets or bolsters…and maybe a couple of yoga blocks…you can also do that standing against the wall so that you’re moving the spine but without weight on the knees or the wrists…” (T3)
2b	“For arthritis patients, I think the yoga that works is therapeutic yoga, that doesn’t strain their joints and it’s unachievable in a group session because the students have to do the practice based on what the teacher is designing. So for me, for arthritis, one-to-one yoga is the best option…” (Th1)
2c	“I think I’m fairly confident because I always check how the person’s feeling, because arthritis can vary daily…so I’m mindful of the weather, the climate, how people are feeling on the day…” (T13)
2d	“…ultimately the student is in control…my students should be comfortable enough to know to stop or put their hand up and say, “No I can’t,”…so I’m confident as long as I can trust them to be confident to tell me then yeah, they’re not going to come and do any harm…” (Th2)
2e	“…I think it’s being more as a facilitator…sometimes their confidence has been knocked, or there’s a lot of the frustrations…with the social aspect and that disabling aspect.” (Th3)
2f	“Yoga practice will only work if the patient is active in their healing so I can design as many practices as I want, if the patient doesn’t go and do those practices at home regularly, nothing will work. So it’s like a partnership between me and the patients.” (Th1)
3a	“A regular exercise class because it’s too fast, too many people, too difficult…they don’t like the gym atmosphere, some people tell me they prefer to be in more, different space, not so many people there…” (T8)
3b	“…I think this idea of having a little bit more appreciation of that idea of befriending and being compassionate towards the health condition…” (Th3)
3c	“…improved function, better movement of joints, feeling more mobile, being able to perhaps walk further or to feel a bit stronger…” (T2)
3d	“…when you sleep better you’re so much more able to manage your pain and again for the mental health benefits that breathing and meditation give you, it’s respite from the pain.” (Th2)
3e	“It can be very isolating not having the full mobility and people will talk to each other, so I think that shared experience is really helpful for people…” (Th3)
3f	“…nobody’s looking to come to yoga as a cure for arthritis, but people are looking to yoga for ways to help improve how they experience their arthritis on a daily basis.” (T14)
3 g	“…I would never say to somebody, “Oh don’t bother taking your medication or taking what the doctor has prescribed” and using yoga in isolation. I think it’s a good tool as an additional part of a healthcare routine…” (Th3)
4a	“…motion is lotion, lubricating the joints keeping things moving and keeping the muscles flexible as well and strengthening the joints…” (T3)
4b	“The warm-up one, Sukhasana with props, Balasana, so going forward again with props, towels and things under them…we do twists but with variations in where the leg is placed, so by the side of the thigh or taking it over, depending on how the hip joints are feeling…we do Trikonasana, Virabhadrasana I, II, Padahastasana, the forward bend, Dhanurasana, Setu Bandhasana, do a lot of Cat and a lot of strength work…” (T10)
4c	“I think mindfulness of the breath is very helpful…focusing on the breath, meditation practises and relaxation are very important to help de-stress people and if they’ve got an inflammatory condition then the relaxation helps to balance the autoimmune system because…when people get over-stressed then that’s going to heighten the sympathetic nervous system, whereas the relaxation then brings and strengthens the parasympathetic response to calm everything down…” (T3)
4d	“…I’d tend to use Ujjayi and think about working with the exhalation, Nadi Shodhana, Anuloma Ujjayi, inhale both nostrils, exhale one…” (T2)
4e	“…the breath work, the meditation, releasing the stress and the anxiety of life and to relax, understanding how to go within, listen to yourself and then once you’ve understood those stepping stones then the asanas can be very useful to arthritis…” (Th4)
5a	“It becomes social as well as exercise and I promote the non-competitive aspect of yoga that the only competition is against your own body…” (T11)
5b	“…there’s a crucial element of a therapeutic relationship that’s built between the therapist and them, where they feel safe, they feel non-judged…” (Th1)
5c	“…the students need to be seeing a therapist who will design a practice which is based on their needs and capacity and takes into account all the other factors that are causing the disease for them…” (Th1)
5d	“…I’m there to help him understand his mind, to understand how to calm down, understanding that it’s okay to be not okay…being able to accept and just not concentrate too much on other people.” (T16)
6a	“…a lot of arthritic people find it quite difficult to get started in the morning…” (T7)
6b	“…arthritis patients have not just got arthritis, they’ve also got diabetes, they’ve got hypertension, they’ve got XYZ…so the logistics of coming to another appointment sometimes becomes a challenge for them.” (Th1)
6c	“…the main challenge is fear through pain…there’s some people I just can’t persuade to kneel, for example. I think that people find it hard to trust their bodies.” (T4)
6d	“…some people would be scared of yoga because I think sometimes yoga has this perception, especially if people look online and see all these lovely thin, young, yogis doing all these headstands and crazy stuff…” (T6)
6e	“The biggest challenge is people not accepting their limitations…I’ll say, “we’re going to come down into a slow squat, any problems with your knees, just sit down” and they will still try and do a squat, even though they’ve been told they shouldn’t be doing it…” (T15)
6f	“…I think the challenge is around promotion to the right groups of people…so possibly if we could link in with maybe organisations like Versus Arthritis or other charities or other chronic pain groups and link in more with health professionals, I think that would help to increase accessibility…” (T14)
6 g	“…most GPs are not going to be recommending yoga to people with pain, which is a real shame.” (T13)
7a	“I think because it’s an unregulated industry…there’s a perception of people that come to yoga classes that the yoga teacher is trained to a particular level or in a particular way…there is a need for regulation of the industry, because we are working with some quite significant health issues…so, just in terms of the safety aspect.” (T13)
7b	“I’m not confident that there’s much yoga research out there because it’s all traditional books of somebody from a long time ago says that this pose will be good for this body part. If there was a scientific document talking about it, I might then look into it if I had people with that issue.” (T5)
8a	“I did a personal trainer qualification and then I did a supplement to that, which was called Teaching Fitness for Long Term Conditions, so that did cover osteoarthritis and rheumatoid arthritis…” (T4)
8b	“…we didn’t look specifically at conditions to fix because it’s much more about internal, the client having that inner feeling and wisdom…if you’ve got, it’s about listening into your own body, so it was less about this is what you would do for this because everyone’s different…” (Th2)
8c	“Understanding the differences between the two conditions and understanding the causes and the effects and then going through the modifications that you can make it safer and enjoyable for the person…” (T7)
8d	“…I would like more hands-on work, where I can stretch someone’s back and we’d do it very gently and decide that works for their body…” (Th2)
8e	“…there’s probably more room for us to learn more about how to adapt yoga to suit people’s mental health needs…” (T14)

## Awareness and clinical knowledge of attendees’ health conditions, including arthritis, and its treatment

### Awareness of health conditions

Yoga providers generally gathered information from the attendees about any health issues they had when attendees joined their sessions for the first time, verbally, or through a written health questionnaire. For those with arthritis, being aware of the affected joints and if they have had any surgery (e.g., hip replacement surgery) was perceived to help identify the specific yoga practices that might be contraindicated, thus ensuring the attendees’ safety (1a). Yoga providers reported a wide range of health conditions disclosed by the attendees including diseases of the eye, endocrine, nutritional, and metabolic diseases (e.g., diabetes), diseases of the circulatory system (e.g., hypertensive diseases, heart diseases), and diseases of the musculoskeletal system, which included arthritis, clinically diagnosed or undiagnosed (1b). Many yoga providers mentioned that some attendees expected joint issues due to ageing and assumed it to be arthritis (1c).

### Clinical knowledge of arthritis, its treatment, and limitations

Yoga providers were fairly knowledgeable about arthritis including its types, signs and symptoms, causes, and mechanisms contributing to joint damage in major types (1d). They mentioned that their attendees with arthritis were generally females aged 50 or over with painful, stiff, inflamed joints and limited mobility being the most commonly reported symptoms. They reported that attendees with a clinical diagnosis of arthritis in the knees, hips, or spine joints generally had osteoarthritis, and joints in the wrists and fingers were typically affected by rheumatoid arthritis. Yoga providers highlighted the “vicious cycle” in arthritis in which people undertake less movement due to persistent pain which further worsens the condition. Some yoga providers knew about the treatment strategies offered by the National Health Service (NHS) for arthritis and their limitations e.g., medications and surgical treatments, their side effects, and NHS physiotherapy service and related concerns (1e). They generally described NHS’s arthritis treatment approach as more “reactive” than “proactive” i.e., predominantly medication-oriented to control symptoms, rather than focusing on providing lifestyle advice or non-pharmacological treatment for symptom prevention (1f).

## Experiences in delivering yoga to attendees with arthritis and their perceptions of arthritis treatment shaped by these experiences

### Experiences of modifying yoga for attendees with arthritis

Yoga providers were aware of how arthritis manifests in individuals differently at different times. So, they emphasised that it was important to encourage attendees with arthritis to “listen to their bodies” and offered modifications (e.g., using props) based on feedback from their attendees and health questionnaires (2a). Yoga teachers shared their experiences of how they tried to keep their sessions inclusive by modifying yoga using a risk-averse approach to meet the needs of all attendees, including those with arthritis. Yoga therapists, however, strongly preferred delivering modifications that were “goal-focused” i.e., catering to what the individual wants to achieve depending on their needs and capabilities in one-to-one sessions (2b).

### Perceived confidence level in delivering yoga to attendees with arthritis

Most yoga providers were reasonably confident in delivering yoga as they were aware and observant, trying to pre-empt the fluctuating needs of attendees (2c). Some of them mentioned that they were confident in delivering gentle movements as long as the attendees took control of their movement and did not push beyond their capabilities (2d). Some yoga providers expressed challenges in dealing with arthritis-specific medical queries of the attendees.

### Perceived importance of their role in arthritis treatment

Most yoga providers felt their role was important in supporting and empowering their attendees with arthritis to take charge of their condition and ensure their overall well-being in arthritis (2e). Yoga therapists, in particular, perceived their role in arthritis treatment was important only if attendees were consistent in their yoga practice and engaged with the yoga therapist through regular feedback to seek adequate support (2f).

## Perceived benefits of yoga for attendees with arthritis

### A better alternative for movement compared to other forms of physical activity

Yoga providers shared that many attendees with arthritis enjoyed and appreciated yoga practice over exercise or gym workouts, as they were looking for a slow-paced, quieter, safer, comfortable, and beneficial alternative for movement (3a). They felt that the “subtle” aspect of yoga enabled the attendees to explore their deep intentions, serving as an escape from daily life stresses, which was not generally the case for exercises or workouts. They also appreciated that yoga practice generated a sense of “acceptance” of their bodies and compassion towards their condition enabling them to focus on what they could do in yoga (3b).

### Physical and mental well-being

Yoga providers spoke about positive feedback received from their attendees with arthritis regarding physical health including relief from painful, stiff, and inflamed joints, reduced muscle tension, increased flexibility, and improved mobility (3c). Yoga providers mentioned that practising yoga diverted attendees’ minds from constant pain and stiffness in joints, making their perception of their symptoms less disruptive, thus reducing stress, and improving mood, eventually contributing to mental well-being (3d).

### Social well-being

Yoga providers highlighted that attendees with arthritis were mostly older and may often feel isolated due to their pain and lack of mobility. Therefore, engaging in yoga practice with fellow attendees with arthritis was perceived to provide an opportunity to forge social connections and increase social interactions with their peers and yoga providers, generating a sense of “community” (3e).

### Potential role of yoga as a part of arthritis treatment

Yoga providers discussed that many attendees practised yoga to improve their ways of dealing with arthritis on a day-to-day basis by maintaining the benefits in their daily lives “off the mat”, i.e., even after the sessions were over (3f). Many yoga providers generally agreed that given its multitude of benefits, yoga could play an additional role in arthritis treatment, alongside other treatments, making it easier for the attendees to cope with their condition (3g).

## Perceived beneficial components of yoga in arthritis treatment

### Yogic poses help mobilise the joints

Yoga providers often highlighted the importance of practising “gentle” or “micro” movements” in yogic poses to lubricate and strengthen the joints and keep the muscles flexible, which was perceived to relieve pain and discomfort in arthritis (4a). Some yoga providers elaborated and some even demonstrated the yogic poses that they thought were helpful in arthritis and delivered in their sessions such as Sukhasana (easy pose), Balasana (child’s pose), Padahastasana (forward bend pose), and Virabhadrasana (warrior pose) (4b).

### Breathing practices and meditation and relaxation practices work together to release mental stress

Yoga providers mentioned that breathing with complete awareness i.e., experiencing the temperature of the breath, deepening the inhalations and exhalations, and noticing the changes in the body consciously through meditation and relaxation practices, seemed to help the attendees with arthritis by distracting their minds from persistent pain, releasing mental stress, and generating a “calming effect” (4c). Several yoga providers mentioned delivering Ujjayi pranayama (victorious breath) and Anuloma Viloma pranayama (alternate nostril breathing) which were perceived to be helpful in arthritis (4d).

### Yogic poses, breathing practices, and meditation and relaxation practices are interrelated in imparting mind–body benefits in arthritis

Yoga providers generally mentioned yoga to be a holistic practice i.e., the interplay between its components seemed crucial to experiencing mind–body benefits in arthritis. Focusing on the breath, while being aware of the body and mind and feeling relaxed was perceived to encourage the attendees to practice yogic poses at their own pace (4e).

## Perceived importance of the yoga environment and yoga provider qualities in arthritis treatment

### Creating and maintaining a positive environment

Yoga providers expressed that it was important to create a “safe and supportive space” for the attendees with arthritis in the yoga sessions, where they could practice yoga without feeling judged about their unique needs and capabilities (5a). Yoga therapists highlighted that it was important for them to establish a “therapeutic relationship” with their attendees with arthritis so that they could trust their yoga therapist and comfortably practise yoga (5b).

### Yoga provider qualities

Yoga providers were passionate about meeting the needs of the attendees with arthritis as they talked about the importance of being empathetic and compassionate towards their attendees’ needs (5c). They shared that it was important to encourage their attendees to accept their physical limitations and enable them to practise yoga with a non-competitive approach (5d).

## Attendee-level concerns perceived as challenges for yoga delivery in arthritis treatment

### Logistical challenges

Yoga providers had a common consideration regarding the timing of yoga sessions as attendees with arthritis tended to be more limited physically in the mornings, so yoga sessions conducted later in the day might be better suited for them (6a). Another concern raised was maintaining a warm temperature at the yoga venues to help the attendees with arthritis keep warmth in their inflamed joints. A yoga therapist noted that most of her attendees with arthritis were older with multiple health conditions and therefore prioritised medical appointments for other health conditions and often missed their yoga therapy sessions for arthritis (6b).

### Apprehensions about trying yoga among attendees with arthritis

Yoga providers expressed their difficulty in motivating attendees with arthritis, especially older attendees, to get them to try yogic poses, due to their fear of worsening pain (6c). Some yoga providers felt that attendees with arthritis were hesitant to practice yoga as they were influenced by the inaccurate and unrealistic media representation of what yoga is, e.g., people with lean bodies practising complex poses such as Sirsasana (headstands) (6d).

### Lack of acceptance of physical limitations among attendees with arthritis

Yoga providers shared that attendees with arthritis were reluctant to accept their physical limitations and ended up practising yogic poses that might be beyond their physical capabilities at the time of practice (6e).

### Limited awareness of yoga among attendees with arthritis

Yoga providers highlighted that there was limited awareness of yoga among those with arthritis leading to a sense that yoga may not be suitable for them. Hence, yoga providers suggested that adequate promotion of yoga through healthcare professionals and relevant organisations supporting people with arthritis might help increase awareness and accessibility to yoga for people with arthritis (6f, 6g).

## System-level concerns perceived as challenges for yoga delivery in arthritis treatment

### Unregulated nature of yoga delivery

Most yoga providers highlighted the lack of regulation of yoga delivery in the UK as a common concern. They spoke about how this meant that yoga sessions might be delivered by those having no formal yoga training, making yoga practice unsafe for attendees with arthritis resulting in injuries. Therefore, yoga providers suggested that yoga delivery should be regulated for the credibility of the yoga providers and the attendees’ safety (7a).

### Limited available scientific evidence on the use of yoga in arthritis treatment

The yoga providers spoke about referring to the available traditional yoga resources for delivering yoga to people with arthritis. Some of them explored published research on the use of yoga for arthritis to gain or update their knowledge (e.g., ideas on making modifications to yogic poses) and found limited scientific evidence. Thus, they highlighted the need for more scientific evidence on using yoga for arthritis treatment (7b).

## Professional yoga training obtained and perceived yoga training needs in arthritis treatment

### Professional yoga training obtained

All yoga providers obtained general yoga teacher training which included some training on delivering yoga for joint conditions (not necessarily specific to arthritis) and was focused on helping the attendees make “gentle” movements. A few of them completed additional training courses, which incorporated details on arthritis types (8a) and aspects of yoga that can be done in seated positions (e.g., chair yoga) for people with arthritis. Four yoga teachers acquired specialised yoga therapy training to become yoga therapists. Yoga therapists emphasised that their yoga therapy training was not specific to a health condition, and instead equipped them with the tools of yoga to meet the attendees’ unique health needs in any health condition, including arthritis (8b).

### Perceived yoga training needs in arthritis treatment

Although the information provided during yoga teacher training was adequate to deliver yoga sessions for any health condition, including arthritis, yoga providers were keen on further arthritis-specific training using the best available scientific evidence. They suggested specialised training such as continual professional development (CPD) courses or hourly workshops would be helpful to gain or update their knowledge on arthritis and deliver safer modifications to yoga practices based on attendees’ unique needs and capabilities in arthritis. They suggested that the specialised yoga training could include the pathophysiology of different types of arthritis and evidence-based information on the yoga practices that might be helpful in arthritis, strategies to provide hands-on modifications to these yoga practices, the rationale behind those modifications, and contraindications if any (8c, 8d, and 8e).

## Discussion

This study provided novel insights into the clinical knowledge, experiences, and perceptions of yoga providers in delivering yoga to attendees with arthritis in the UK. We found that the yoga providers were fairly knowledgeable about arthritis, its types, signs, symptoms, causes, mechanisms, and treatment. They were reasonably confident in delivering yoga to attendees with arthritis and perceived their role to be significant in supporting these attendees by helping them take charge of their condition. They perceived yoga to have many interrelated mind–body benefits in arthritis, which included physical, mental, and social well-being. They highlighted the importance of a safe and supportive environment in the yoga sessions, where each attendee feels comfortable practising yoga without being judged about their individual needs in arthritis. They were cognisant of the lack of awareness of yoga among attendees with arthritis and their apprehensions about trying yoga for their condition. Their major challenge was the lack of regulation of yoga delivery in the UK. The availability of scientific evidence on the use of yoga for arthritis was also generally felt to be limited by the yoga providers. However, they suggested that accessibility to further arthritis-specific yoga training provisions using the best available scientific evidence would be helpful in arthritis treatment.

Yoga providers in our study perceived that yoga was helpful in arthritis as it could be modified using a gentle approach to suit the attendees’ changing needs and capabilities. The adaptability of yoga to individual needs was found to be helpful, especially for people who generally tend to be physically limited due to musculoskeletal conditions, including arthritis, as suggested by findings from a systematic review [35]. Our findings highlighted yoga providers’ perceptions of the interlinked mind–body benefits of yoga imparting overall well-being in arthritis. Existing literature suggests that yoga practice tends to increase body awareness, reduce pain perception and mental stress, and improve overall well-being [36, 37]. Social support conferred by yoga practice was identified by yoga providers in our study to be helpful for attendees with arthritis, as it made them feel socially connected. Similarly, a qualitative study exploring yoga’s impacts on individuals’ social well-being highlighted how yoga practised in groups helped increase positive social interaction and overcome isolation [37–39]. Underlining yoga’s wide range of perceived benefits, our findings add to evidence suggesting that yoga could have a role in alleviating arthritis symptoms in addition to standard treatment [40–43].

A key theme in our study described yoga providers’ perceptions of how yoga is delivered. Delivering yoga in a safe and supportive space and the yoga providers’ ability to be responsive and compassionate towards attendees’ needs were perceived to be crucial in arthritis treatment, reinforcing the importance of the yoga environment and the yoga provider traits from previous qualitative research [39, 41, 44–46]. Another salient finding from our study highlighted yoga providers’ concern that with no existing regulations for yoga delivery in the UK, undertrained individuals can deliver yoga to people with or without any health condition. In addition, the variability in practices delivered as “yoga” might be contraindicated in specific health conditions, including arthritis, leading to yoga-related injuries. These perceived challenges underpin the need for effective regulation and accreditation of yoga delivery in the UK to ensure the credibility of yoga providers and the safety of the attendees, as highlighted previously by yoga providers [47]. Yoga providers shared that some attendees with arthritis thought yoga was a physically challenging practice and were therefore reluctant to try yoga. Yoga providers added that unrealistic media portrayals of yoga might be a reason for their fear of practising yoga. This was supported by existing qualitative evidence suggesting that yoga was not generally represented as an adaptable and inclusive practice for varying needs and capabilities in the media, affecting its promotion and awareness among people [48].

To maximise yoga’s potential in arthritis treatment, there is a need to increase awareness of yoga and its benefits and promote its authentic representations to encourage people with arthritis to practice yoga. This could include initiatives to endorse yoga programmes for arthritis through partnerships between healthcare professionals, professional yoga organisations, and arthritis care organisations in the UK. Further, the provision of arthritis-specific yoga training programmes that are based on the best available scientific evidence is needed for yoga providers to meet the needs of attendees with arthritis adequately [49]. In addition, future research exploring qualitative insights from people with arthritis practising yoga will be helpful for triangulation with our findings to get a deeper understanding of the use of yoga in arthritis treatment.

To our knowledge, this was the first study to explore clinical knowledge, experiences, and perceptions of UK-based yoga providers in arthritis treatment. Our analysis of UK-based yoga providers’ accounts reflects the potential transferability of these findings to similar contexts and healthcare settings. Although all the interviews were conducted online, the lead researcher established rapport with each participant through an informal and friendly conversation before the interview and using positive non-verbal behaviours (e.g., eye contact, nodding) during the interview. Qualitative research is largely dependent on the skills of the researchers, therefore making it challenging to maintain, assess, and demonstrate methodological rigour. The lead researcher had no prior acquaintance with the participants and was not familiar with yoga practice in the UK but had some experience as part of her formal education in India. All the senior researchers involved in the analysis had significant experience in evidence-based healthcare research; one of them had an educational background in Ayurveda (including yoga) from India.

Reflexivity was maintained throughout the data collection and analysis, with the researchers actively reflecting on their assumptions, backgrounds, beliefs, and experiences to minimise potential biases and ensure a balanced interpretation of the findings. Furthermore, selection bias may have been introduced as yoga providers with a strong interest or positive views on yoga’s role in arthritis treatment may have volunteered to participate. Response bias could have further influenced our findings, with yoga providers possibly providing socially desirable responses, leading to an overrepresentation of positive insights. For instance, when asked about challenges in delivering yoga to attendees with arthritis, most yoga providers stated that no yoga-related injuries had occurred in their sessions, even though some injuries may have actually taken place. This study primarily represented yoga teachers’ views and since yoga in the UK is predominantly delivered by these professionals [14], their insights would be crucial in identifying and addressing the challenges in delivering yoga in arthritis treatment.

## Conclusion

Yoga providers felt they could play a key role in arthritis treatment in the UK, provided yoga is adequately promoted and made accessible to people with arthritis, yoga delivery is regulated, and yoga providers have access to arthritis-specific yoga training using the best available scientific evidence.

## Data Availability

The data supporting the findings of this study are available from the corresponding author upon reasonable request. A deidentified data set will be available upon request unless there are legal or ethical reasons for not doing so.

## Declarations

### Related congress abstract publication

The study abstract has neither been accepted nor published as a conference abstract.
